# Intravenous leiomyomatosis with inferior vena cava or intracardiac extension and concurrent bilateral multiple pulmonary nodules: A report of 2 cases

**DOI:** 10.1097/MD.0000000000004722

**Published:** 2016-09-02

**Authors:** Guorui Zhang, Xin Yu, Jinghe Lang

**Affiliations:** Department of Obstetrics and Gynecology, Peking Union Medical Collage Hospital, Chinese Academy of Medical Sciences, Beijing, China.

**Keywords:** benign metastatic leiomyoma, intravenous leiomyomatosis, pulmonary nodules

## Abstract

**Background:**

Intravenous leiomyomatosis is a special type of uterine leiomyoma and features formation and growth of benign leiomyoma tissue within vascular wall. Benign metastatic leiomyoma refers to benign leiomyoma metastasizing to extra-uterine sites, dominantly lung. Solitary or multiple small nodules in the lung can be seen in image scans.

**Methods:**

We report 2 cases of intravenous leiomyomatosis with inferior vena cava or intracardiac extension and concurrent multiple nodules in bilateral lungs.

**Results:**

Case 1 was a 40-year-old woman with a large mass in pelvic cavity, masses in heart chambers, and disseminates pulmonary nodules detected at preoperative image scans. Masses in pelvic cavity and heart were resected in a 2-stage surgery. Histology examination confirmed the diagnosis of intravenous leiomyomatosis. Pulmonary nodules stayed stable during follow-up. Case 2 was a 37-year-old woman with 3 times of uterine-related surgeries. A pelvic mass appeared again and filling defect was observed in left ovarian vein, right renal vein, right common iliac vein, and inferior vena cava. Tumors in pelvic cavity and within vessels were removed in a 1-stage surgery. Histology examination confirmed the diagnosis of intravenous leiomyomatosis. Pulmonary nodules remained stable during follow-up.

**Conclusion:**

The incidence of benign metastatic leiomyoma in patients with intravenous leiomyomatosis might be relatively high. Metastasis of intravenous leiomyomatosis lesions was a possible source of benign metastatic leiomyoma in these cases.

## 1 Introduction

Intravenous leiomyomatosis (IVL) is a special type of uterine leiomyoma and features formation and growth of benign leiomyoma tissue within vascular wall. The tumor can grow along blood vessels, extending to iliac vein, inferior vena cava, and even heart.^[1]^ IVL with inferior vena cava or intracardiac extension is a rare disease and has been reported in approximate 200 cases. Benign metastatic leiomyoma (BML) refers to a benign leiomyoma metastasizing to extra-uterine sites, dominantly lung. About 60 cases have been reported in the literature.^[2]^ Nodules in 70% of all cases are disseminated in double lungs, with a mean number of approximately 6 and a mean diameter of 1.8 cm.^[3]^ Solitary or multiple small nodules in the lung can been seen in image scans. Both IVL with inferior vena cava or intracardiac extension and BML are uterine smooth muscle tumors with unusual growth patterns.^[4]^ Herein, we report 2 cases of IVL with inferior vena cava or intracardiac extension and concurrent multiple nodules in bilateral lungs.

## 2 Cases report

### 2.1 Case 1

**Figure 1:**
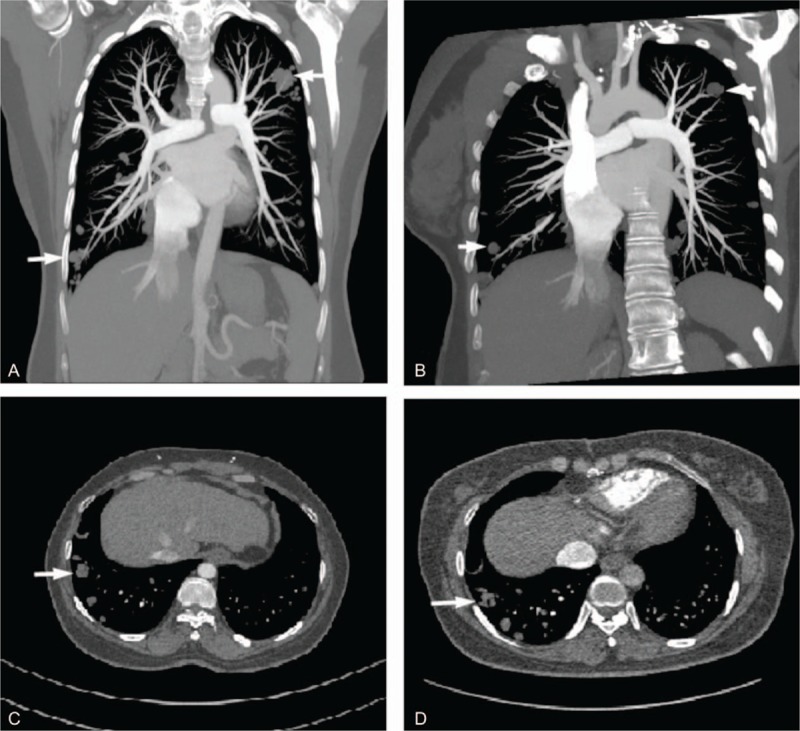
Computed tomography images of case 1 were shown. Multiple round-shaped nodules (arrows) with different sizes were randomly distributed in bilateral lung fields in coronary plane (A), sagittal plane (B), and axial plane (C) in April 2014. Multiple nodules in bilateral lung fields changed little (D) in September 2014.

The patient was 40 years old, gravid 2, and parity 1. Pelvic ultrasonography during routine physical examination in 2009 revealed a mass sized 2 cm within uterus. In June 2011, the patient presented frequent micturition, 8 to 12 times/d. Pelvic ultrasonography revealed the uterine mass enlarged obviously, with a diameter of 10 cm. The patient rejected surgery till 10 months later. Computed tomography (CT) of pelvic cavity in April 2014 showed a 7.0 × 4.7 cm sized, irregular shaped, unclear boundary, and soft tissue density mass within uterus. CT pulmonary angiography and CT venography suggested a strip of filling defect in right ventricle and right atrium, partially overlapping the tricuspid valve. Multiple round-shaped nodules of different sizes were randomly distributed in bilateral lung field (Fig. 1A–C). Echocardiography showed right ventricular enlargement, a mass sized about 4.4 × 3.0 cm on tricuspid chordae tendineae, and moderate tricuspid insufficiency.

On June 30, 2014, intracardiac mass resection and tricuspid valve plasty, a first-stage operation, were performed. During the surgery, a total of 5 ovoid, gray-white colored, tough texture masses were removed. The masses were in different sizes and the largest diameter was 3 cm, with a pedicle clinging to chordae tendineae and papillary muscles. Histologically, the masses presented spindle-like smooth muscle cells without atypia, companied with local mucus and hyaline degeneration. Immunohistochemical stain showed desmin (+), smooth muscle actin (SMA) (+), S-100 (−), estrogen receptor (ER) (+), and progesterone receptor (PR) (+). Anticoagulation treatment with warfarin and gonadotropin releasing hormone agonist (GnRHa) therapy were administered for 6 months. On December 24, 2014, the patient received a second-stage operation, laparoscopic hysterectomy, and bilateral oophorectomy. During the operation, the uterus was as large as 8 weeks’ pregnant uterus, and there were multiple cystic masses on the right side of uterus, sized about 6 × 3 cm, spreading into right iliac vessels. Complete resection of tumors was achieved. Histological and immunohistochemical features were same as masses removed from heart chambers. The final diagnosis was IVL with intracardiac extension. And pulmonary nodules were suspected as pulmonary BML, unnecessary for further treatment. The patient rejected postoperative hormonal treatment. No signs of recurrence were observed and pulmonary nodules stayed stable (Fig. 1D) in 2 months’ follow-up (then lost to follow up).

### 2.2 Case 2

**Figure 2:**
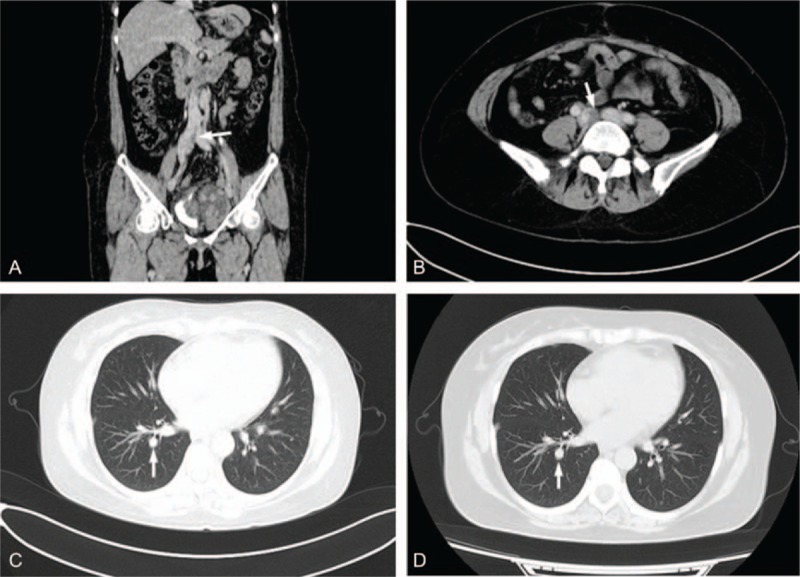
Computed tomography images of case 2 were shown. A pelvic mass and filling defect right common iliac vein and inferior vena cava (A and B) were detected in March 2014. Multiple round-shaped nodules were randomly distributed in bilateral lungs in March 2014 (C). Multiple nodules in bilateral lungs remained stable in April 2015.

The patient was 37 years old, gravid 1, and parity 1. She received hysterectomy and right oophorectomy on condition of uterine leiomyoma at age of 33. Two months later, pelvic mass recurred on ultrasonography. And another surgery resecting broad ligament tumors was performed with a pathological diagnosis of broad ligament leiomyoma. One year ago, pelvic ultrasonography revealed a mass with a diameter of 8 cm in left pelvic cavity. The patient did not search for treatment. Three months ago, the patient complained of intermittent abdominal pain, and another surgery was performed to remove the pelvic mass. The pathological diagnosis was pelvic multiple leiomyoma. At 2 months’ postoperative follow-up, CT revealed a left pelvic mass with the maximum diameter of 5.3 cm, without enhancement. Filling defect was observed in left ovarian vein, right renal vein, right common iliac vein, and inferior cava (Fig. 2A and B). Besides, multiple round-shaped nodules were found randomly distributed in bilateral lung fields (Fig. 2C).

On June 4, 2014, the patient underwent resection of inferior vena cava tumor, pelvic mass, and left oophorectomy. During the operation, transesophageal echocardiography revealed that the tumor was floating in the vascular cavity. The distal end of tumor was located at the entrance of the right atrium, without intruding into right atrium. Postoperative pathology confirmed the diagnosis of IVL and immunohistochemical stain featured Desmin (+), SMA (+), CD117 (−), ER (+), and PR (+). Tumors in vessels and pelvic cavity were resected completely. Nodules in bilateral pulmonary were clinically suspected as pulmonary BML. Six days after the surgery, serum follicle stimulating hormone level was 29.18 IU/L, and serum estrogen 2 level was 37 pg/mL.

On October 10, 2014, CT scans revealed multiple mixed density masses in pelvic cavity, and the largest locating on the left side of bladder sized 12 × 7.2 cm. Filling defect appeared again in right iliac vein, right common iliac vein, and lower part of inferior vena cava. Then GnRHa therapy was administered for 6 months. In April 2015, CT scans showed pelvic mass with a size of 12.9 × 6.0 cm, and intravascular filling defect remained stable. Multiple nodules in bilateral lungs changed little in the process (Fig. 2D).

### 2.3 Ethical statement

The report was ethically approved by the Institutional Board of Peking Union Medical College Hospital. Informed consent was obtained from both the patients.

## 3 Discussion

Both IVL and BML were leiomyomas with benign histological characteristics, no abnormal mitosis and no atypia, but they had special growth patterns.

The pathological mechanism of the IVL was not clear, and currently 2 explanations were existing. One is the Knauer theory, which believed IVL originated from vascular wall.^[5]^ The other is the Sitzenfry theory, which believed IVL occurred when uterine leiomyoma invading blood vessels.^[6]^ In addition, there were 3 hypotheses for the pathogenic mechanism of BML. One was that BML derived from uterine myometrium layer. The second theory suggested that BML might be secondary to implantation and proliferation of benign smooth muscle cells, by IVL or mechanical means.^[7]^ The third theory was that BML was a part of systemic leiomyoma, featuring multifocal and independent smooth muscle tissue proliferation.^[8]^

IVL with inferior vena cava or intracardiac extension could present symptoms relating to heart function insufficiency, including lower extremity edema, palpitation, chest tightness, and so on. Surgery was the principal treatment.^[9]^ Total hysterectomy, bilateral oophorectomy, and resection of all visible tumors should be performed in the operation. According to the range of lesions and patients’ tolerance to surgery, 1-stage and 2-stage operations were in option. The postoperative recurrence rate was high, approximately 30%.^[10]^ Incomplete resection was a high-risk factor for postoperative recurrence. Immunohistochemical stain of IVL lesions showed ER, PR positive, and thus antiestrogen therapy was the primary postoperative therapy.^[11]^ However, currently there were no large-scale cohort reports illustrating the outcome of hormone therapy. Hormone treatment had different effect on patients receiving incomplete resection of tumor in previous literature.^[12–17]^

BML was asymptomatic in most cases and was usually discovered by accident. BML showed benign pathological features, with little cell mitosis, and positive for ER and PR.^[18]^ Pathological tests helped to make the final diagnosis. BML should be differentiated from metastasis of various malignant tumors. Treatment methods were determined according to symptoms. Tumors without clinical symptoms and without obvious growth could be monitored at follow-up, requiring no immediate treatment, while tumors growing rapidly or suspected malignant needed treatment. In cases with a solitary nodule, curative surgical excision was indicated. But in cases with multiple nodules, most would be left with residual tumors, and postoperative follow-up was important to prevent major complications caused by residual tumor progression. Antiestrogen hormone therapy demonstrated disease control or regression in 79% patients in previous literature.^[2]^

Multiple pulmonary nodules were found simultaneously with the detection of intravascular lesions in the 2 patients in this report. These nodules, considered as BML, stayed stable in the process. After careful literature review, there were 6 cases of IVL complicated with pulmonary BML in the literature. Among them, IVL lesions and pulmonary nodules were found simultaneously in 4 patients. Lee et al^[19]^ described a case of pathological diagnosed pulmonary benign metastasizing leiomyoma associated with uterine IVL in a 46-year-old woman. Results of comparative genomic hybridization showed lesions in the 2 sites had significantly overlapping complex genomic changes, suggesting that the 2 lesions were closely related to each other. Arif et al^[20]^ reported a case of a 42-year-old patient presenting a left L4 nerve root lesion, a left para-vesical lesion, multiple pulmonary metastases, and an intracava lesion. All those lesions were confirmed histologically to be leiomyoma strongly positive for ER. Matsumoto et al^[21]^ and Koh et al^[22]^ reported another 2 similar cases. Besides, pulmonary nodules were found after IVL diagnosis in 2 patients. Bodner-Adler et al^[23]^ reported a case underwent abdominal hysterectomy because of IVL. Postoperatively, multiple nodules in lungs were detected in the lung scans, and biopsy confirmed the diagnosis of pulmonary BML. Sun et al^[24]^ reported a patient whose multiple pulmonary nodules were found during postoperative IVL follow-up of IVL. The author believed that the pulmonary lesions were BML, although without histological evidence.

## 4 Conclusion

When IVL patients presented bilateral multiple pulmonary nodules, the possibility of BML should be considered, and excessive treatment due to misdiagnosis as malignant tumor metastasis should be avoided. The incidence of BML in patients with IVL might be relatively high. Metastasis of IVL lesions was a possible source of BML in these cases.
